# Case Report: Primary Cardiac T-Cell Lymphoma With Complete Atrio-Ventricular Block Diagnosed by Endomyocardial Biopsy

**DOI:** 10.3389/fimmu.2022.890059

**Published:** 2022-06-16

**Authors:** Panpan Chen, Yuanyuan Hao, Xi Qiu, Xibin Xiao, Wei Zhu, Yang Xu, Wenbin Qian

**Affiliations:** ^1^ Department of Hematology, The Second Affiliated Hospital, College of Medicine, Zhejiang University, Hangzhou, China; ^2^ Key Laboratory for Cancer Molecular Cell Biology, Life Sciences Institute, Zhejiang University, Hangzhou, China; ^3^ Department of Cardiology, The Second Affiliated Hospital, College of Medicine, Zhejiang University, Hangzhou, China; ^4^ Provincial Key Laboratory of Cardiovascular Research, Hangzhou, China

**Keywords:** primary cardiac lymphoma, T-cell lymphoma, complete atrioventricular block, immunotherapy, case report

## Abstract

Primary cardiac lymphoma (PCL) is a rare disease, the definite diagnosis of which is sometimes difficult and mainly relies on endomyocardial biopsy. Primary cardiac T-cell lymphoma (PCTL) is an extremely rare sub-type of PCL. Here, we report on a 47-year-old female with PCTL who presented with fever, syncope, palpitations, and a third-degree atrioventricular block (AVB) on electrocardiogram. Chemotherapy was administered with two courses of methotrexate, cyclophosphamide, liposomal doxorubicin, vincristine, and dexamethasone (MTX-CHOP). As the tumor vanished, AVB changed from third degree to second degree and finally to sinus rhythm. In conclusion, endomyocardial biopsy is valuable in the diagnosis of primary cardiac lymphoma. It is worth noting that alterations in the electrocardiogram may indicate an attack on the heart by PCTL.

## Introduction

Primary cardiac lymphoma is defined as a malignant lymphoma located in the myocardium and pericardium with cardiac symptoms due to myocardial infiltration of the lymphoma as the main manifestation (1). Due to its rarity and difficulty in confirming the diagnosis, it is largely reported as an isolated case. In 2016, Gordon et al. reviewed, in Pubmed, 94 cases between 1990 and 2015 of non-hodgkin lymphoma (NHL) involvement with biopsy evidence (2). Of these cases, only 51 were diagnosed as primary cardiac lymphoma and 43 were diagnosed as secondary. Primary diffuse large B-cell lymphoma was the most common histological subtype (58%), followed by T- cell lymphoma (16%), Burkitt lymphoma (9%), and small lymphocyte lymphoma (6%) (2).

Here, we report a case of primary cardiac peripheral T-cell lymphoma with third-degree atrioventricular block that was successfully treated and reversed to first-degree AVB. Wang et al. also reported a primary heart T-cell lymphoma with third-degree atrioventricular block before treatment, which turned into first-degree atrioventricular block one week later, similar to our case (3).

## Case Presentation

A 47-year-old female patient visited the emergency department of our hospital on 30 May 2020 reporting an episode of syncope lasting 1-2 minutes. Three hours ago, the patient experienced syncope without convulsions, incontinence, vertigo, tinnitus, nausea or vomiting, chest pain, or chest tightness. About three months ago, she started to have a fever up to 38.6°C, accompanied with dizziness and weakness. She was diagnosed with an infection of unknown etiology at another clinic and was prescribed anti-infective therapy. The medication taken included antibiotics and methylprednisolone. However, the anti-infection therapy was ineffective and the fever persisted.

She was then admitted into the Department of Cardiology in our hospital, on physical examination, her heart rate was 59 beats/min, the blood pressure was 100/56mmHg, and respiratory rate was 19/min. The thorax was symmetrical, with no deformities on inspection, both lungs had clear breathing sounds, and neither dry nor wet rales were heard on auscultation. The heart rate was normal rhythm and no pathological murmur was detected in each valve auscultation area. The abdomen was soft, without pain from pressure, and there was no swelling of either lower limbs.

PET-CT examination suggested a thickening of the posterior and bilateral walls of the paranasal nasopharynx and soft tissues, enlarged shape of the left atrium, bilateral pulmonary valves and mediastinum, enlarged multiple lymph nodes in the left clavicular region with increased FDG metabolism, enlarged spleen, and increasingly diffused, uneven FDG metabolism in the bone marrow cavity. Positive infectious disease or possible hematologic lymphoma was considered.

Cranial MRI combined with 3D enhancement and diffusion imaging (3.0T) showed abnormal enhancing shadow in the posterior wall of the nasopharyngeal apex and both walls extending across the middle and posterior skull base to the right temporal and pontocerebellar regions, involving the right temporal and right cerebellar hemisphere meninges, all of which indicated infectious lesions.

**Figure 1:**
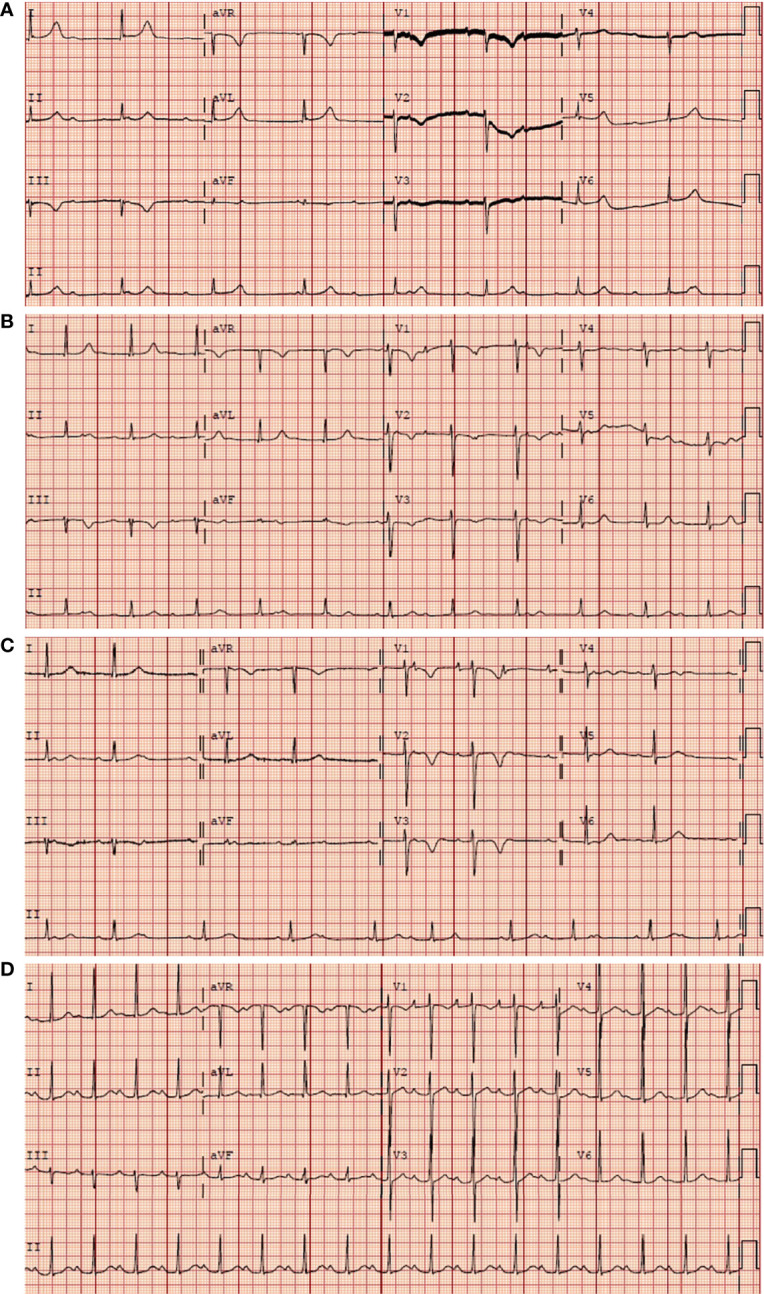
Serial electrocardiographic strips upon patient admission. **(A)** Complete AVB before chemotherapy. **(B)** Still complete AVB on the second day after chemotherapy. **(C)** The complete AVB converted to second degree type 2 AVB on the sixth day after chemotherapy. **(D)** A first degree AVB on the eighteenth day. The initial electrocardiogram (ECG) of the patient during the chemotherapy.

**Figure 2:**
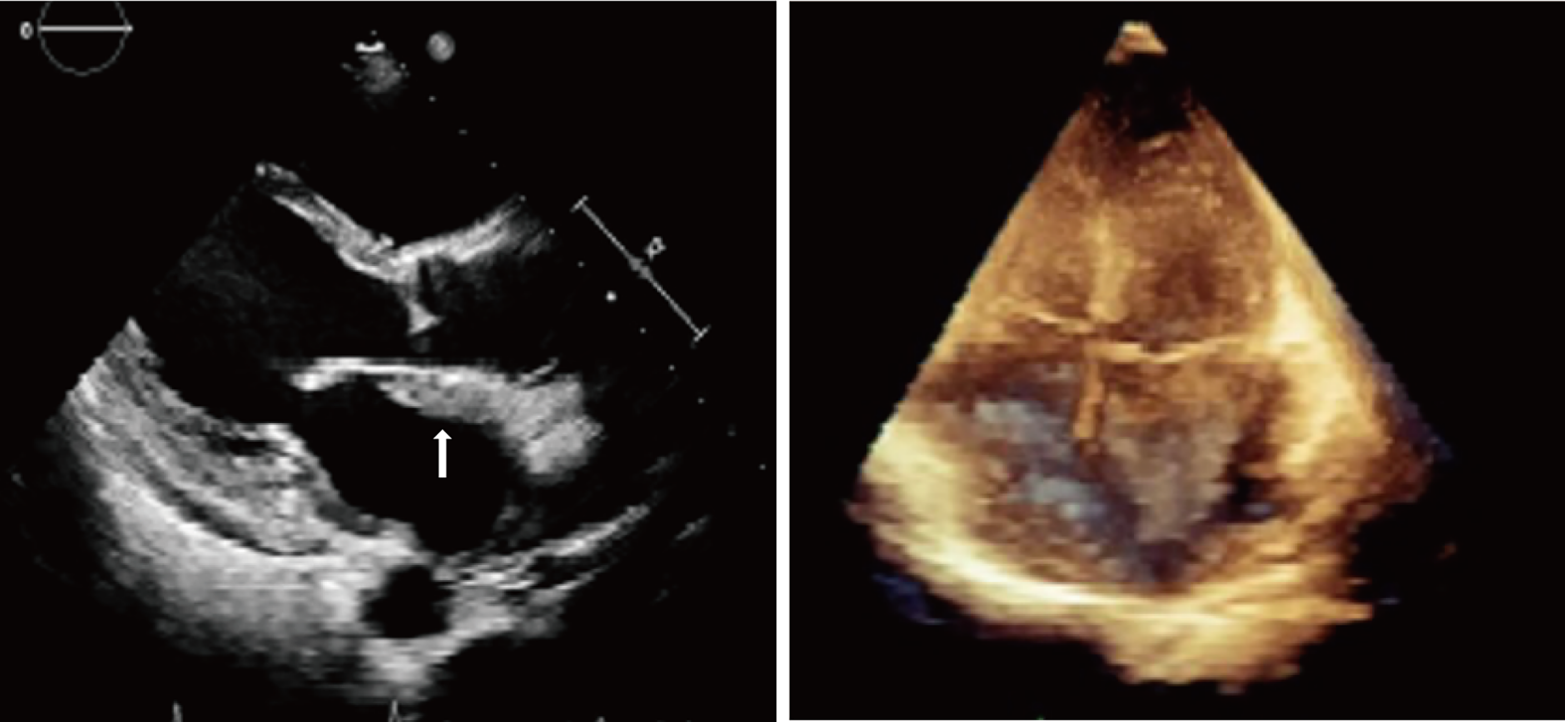
Transthoracic echocardiography showed a homogeneous isoechoic layer thickening of the endocardial surface of the left atrium across the entire left atrial wall.

The initial electrocardiogram (ECG) showed a complete atrioventricular block (AVB) with a junctional rhythm (**Figure 1A**). Blood examination showed troponin levels within normal range and the brain natriuretic peptide precursor level was 1388 pg/ml. Transthoracic echocardiography (**Figure 2**) shows a homogeneous isoechoic layer thickening of the endocardial surface of the left atrium across the entire left atrial wall with a thickness of approximately 1.06 cm, and the same changes were observed in the left auricle. The rest of the structures were indistinguishable. Left cardiac ultrasonography suggested a homogeneous isoechoic filling of the left atrial wall and left auricle with no perfusion in the imaging.

**Figure 3:**
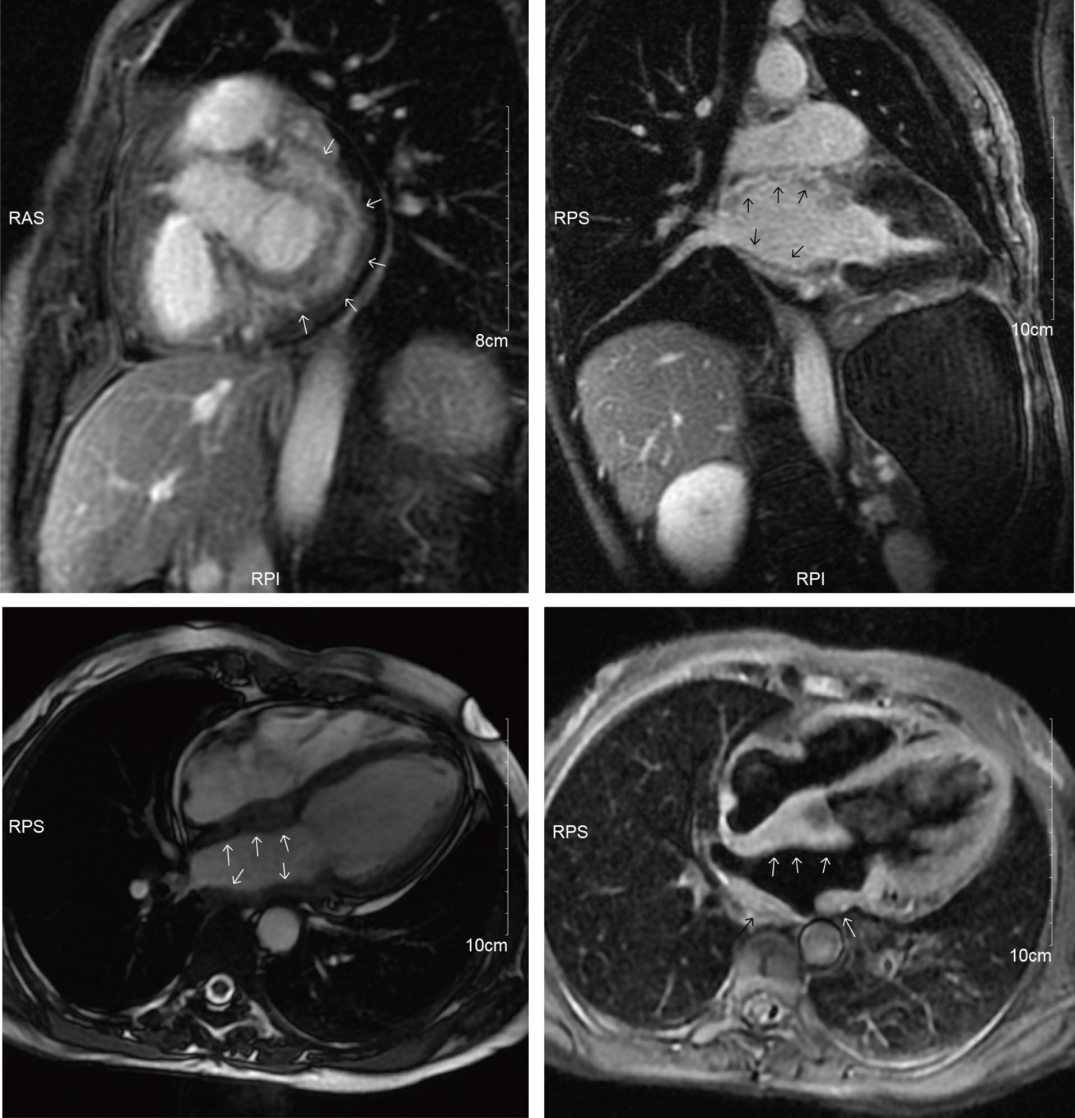
CMR showed a significant thickening of the left atrial wall.

A cardiac magnetic resonance (CMR) examination was then carried out for the patient (**Figure 3**), confirming a significant thickening of the left atrial wall with no enlargement of the left and right atrial chambers and a triple inversion recovery (IR) sequence showing a more homogeneous high signal with significant inhomogeneous enhancement on the delayed scan. There was no hypertrophy of the ventricle, no dilatation or stenosis of the aorta or pulmonary arteries, and the left ventricular ejection fraction was 66%.

Based on the medical history of ineffective anti-infective therapy with homogeneous isoechoic thickening of the endocardial layer across the whole left atrium and auricle, which involved the AV conduction system, an amyloid or tumorgenic abnormality should be suspected. From an etiologic standpoint, a further examination is required to make a clear diagnosis for the patient

**Figure 4:**
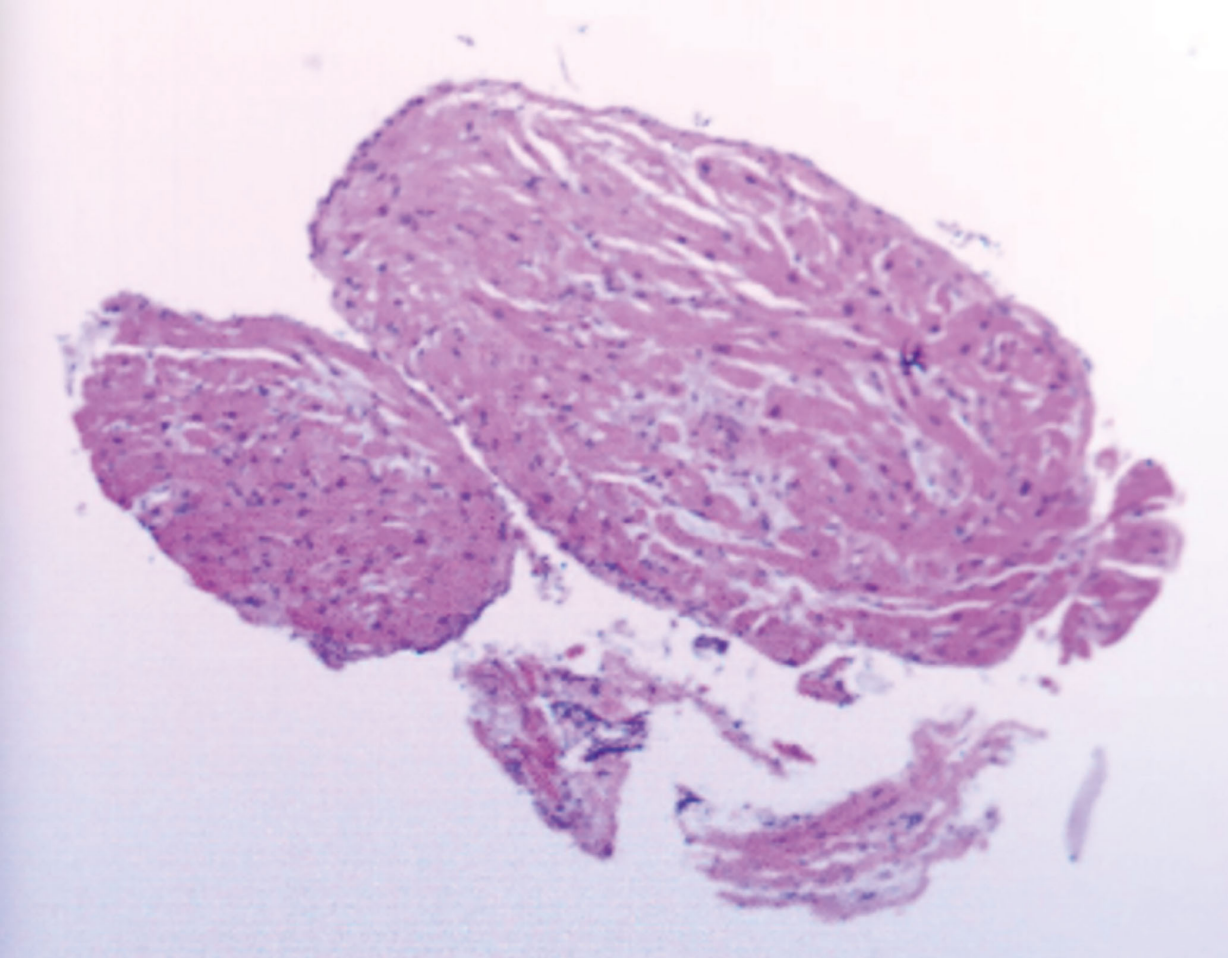
EMB taken from the left atrium showed hyperplasia of myocardial and fibrous tissue with localized irregular lymphoid-like cells.

To clarify the diagnosis, an endomyocardial biopsy (EMB) examination and temporary pacemaker implantation were performed in the catheterization laboratory with the guidance of fluoroscope, with ethical consent from the patient’s family. A histological examination of samples taken from the left atrium suggested (**Figure 4**) hyperplasia of myocardial and fibrous tissue with localized irregular lymphoid-like cells. Immunohistochemical staining showed the tumor cells were diffusely positive for CD3, CD5 and CD8, scattered positive for CD2, CD7, CD4 and Granzyme B, ki-67 proliferation index reached 30%. However, the tumor cells were negative for CD20, CD56, CD10, BCL6, PD-1, CD21, CXCL13, CD30, ALK, CD99 and P53. T-cell-related markers CD3+, CD5+, CD7 individual+, CD2 individual+, B cell-related markers CD20 and CD21 negative, cell proliferation-related markers ki-67 30%+, CD4 individual+/CD8+. Differentiating from angioimmunoblast T-cell lymphoma, this case was negative for CD10, BCL6, PD-1, and CXCL13, and negative for the markers CD30 and ALK associated with anaplastic large cell lymphoma. The above markers demonstrated that the pathological immunophenotype of this case was non-specific peripheral T-cell lymphoma and not other types of lymphoma. Thus, *in situ* hybridization detected tumors negative for Epstein-Barr virus encoded with small mRNA. In combination with immunohistochemistry and *in situ* hybridization of lymph node pathology, this patient was diagnosed with peripheral T-cell lymphoma. The patient was subsequently transferred to the hematology department for chemotherapy.

The patient was treated with MTX+CHOP chemotherapy (methotrexate 1.52g day1, cyclophosphamide 750mg/m^2^ day1, liposomal doxorubicin 30mg/m^2^ day1, vinorelbine 30mg/m^2^ day1, dexamethasone 15mg day1-5). After 2 courses of chemotherapy, efficacy was assessed as complete remission of the patient. ECG showed the atrioventricular block changed from third degree to second degree (**Figure 1B, C**) and finally to sinus rhythm cardiac (**Figure 1D**). Additionally, the ultrasonography showed no significant abnormalities in size, morphology, structure, and functional blood flow of the heart. Electrocardiogram suggested sinus rhythm. No further atrioventricular dissociation was observed and the pacemaker was removed.

## Discussion

The case presented here is a rare case of peripheral T-cell lymphoma of the heart and was diagnosed by EMB. The patient was well-treated and had a remarkable result at the end of the first course of treatment. The patient went from third degree AV block to second degree AV block and finally converted to sinus rhythm. Echocardiographic evaluation at the end of the two courses showed no significant abnormalities in the left atrium.

Primary cardiac tumors are rare, with incident rates ranging from 1.38 to 30 per 100,000 people per year (4). Among primary cardiac tumors, 80% are benign and 20% are malignant (5). Malignancies are classified by tissue type as mesenchymal (sarcoma), lymphoma (lymphoma), and mesothelial (mesothelioma), of which sarcoma is the most common (6). Although 16%-28% of patients with diffuse lymphoma have cardiac involvement, primary cardiac lymphoma is very rare. Among the cardiac malignancies found, cardiac lymphomas are quite common and may involve the heart diffusely (6). Among lymphomas with cardiac involvement, the most common type of pathology is diffuse large B-cell lymphoma (58%), whereas T-cell lymphoma is quite rare (16%) (2) And 62% of patients with peripheral T-cell lymphoma present with extranodal diseases (7). T-cell lymphoma has been reported to involve the skin (8), heart (9), central nervous system (CNS) (10), intestine, and lungs (11). Also, 23% of primary cardiac lymphomas are presented with arrhythmias and AVB is even more rare at 8% (12). The common clinical manifestations of cardiac lymphoma are dyspnea (64%) and pericardial effusion (58%) (13). However, in the present case, the main manifestation encountered was AVB, which emphasizes the value of electrocardiographic changes in the diagnosis of sudden cardiac disease. In fact, the patient was implanted with a temporary pacemaker. As the tumor vanished, so did the AVB, implying that the electrocardiographic changes caused by the lymphoma were reversible.

The CHOP regimen is recommended as a first-line chemotherapeutic regimen for cardiac lymphoma (14). The CHOP regimen has resulted in an overall response rate of about 60% (15) and a median progression-free survival is around 13 months in T-cell lymphoma (16). Although T-cell lymphoma is less common in the CNS, CNS involvement cannot be excluded since biopsy cannot be performed in nasopharyngeal area. Therefore, as there was a concern of CNS involvement, the patient was treated with CHOP regimen accompanied by MTX for the first 2 courses.

EMB is indicated for the diagnosis of intracardiac masses (17) and arrhythmogenic cardiomyopathy (18). In those patients with refractory arrhythmias, it is clinically relevant to perform EMB to evaluate cardiac T-cell lymphoma. Most cardiac lymphomas are of B-cell lineage presenting B-cell markers, such as CD19, CD20, and CD22. In our case, the biopsy of this patient showed CD3+ and CD5+, which were considered typical characteristics of T-cell lymphoma. With help of EMB, this patient was diagnosed and treated properly without delay.

## Conclusion

In this case, we reported a rare case of cardiac T-cell lymphoma, in which EMB was of valuable in diagnose of cardiac lymphoma. Of note, electrocardiography alteration may imply the cardiac assault in T-cell lymphoma.

## Data Availability Statement

The original contributions presented in the study are included in the article/supplementary material. Further inquiries can be directed to the corresponding author.

## Ethics Statement

Written informed consent was obtained from the individual(s) for the publication of any potentially identifiable images or data included in this article.

## Author Contributions

PC and YH collected the data and performed the literature. WQ and YX revised the work. XQ, XX and WZ support-ed the study and reviewed the manuscript. All authors contributed to the article and approved the submitted version.

## Conflict of Interest

The authors declare that the research was conducted in the absence of any commercial or financial relationships that could be construed as a potential conflict of interest.

## Publisher’s Note

All claims expressed in this article are solely those of the authors and do not necessarily represent those of their affiliated organizations, or those of the publisher, the editors and the reviewers. Any product that may be evaluated in this article, or claim that may be made by its manufacturer, is not guaranteed or endorsed by the publisher.
